# Estimated cochlear neural degeneration is associated with loudness hypersensitivity in individuals with normal audiograms

**DOI:** 10.1121/10.0011694

**Published:** 2022-06-13

**Authors:** Kelly N. Jahn, Kenneth E. Hancock, Stéphane F. Maison, Daniel B. Polley

**Affiliations:** Eaton-Peabody Laboratories, Massachusetts Eye and Ear, Boston, Massachusetts 02114, USA kelly.jahn@utdallas.edu, ken_hancock@meei.harvard.edu, stephane_maison@meei.harvard.edu, daniel_polley@meei.harvard.edu

## Abstract

In animal models, cochlear neural degeneration (CND) is associated with excess central gain and hyperacusis, but a compelling link between reduced cochlear neural inputs and heightened loudness perception in humans remains elusive. The present study examined whether greater estimated cochlear neural degeneration (eCND) in human participants with normal hearing thresholds is associated with heightened loudness perception and sound aversion. Results demonstrated that loudness perception was heightened in ears with greater eCND and in subjects who self-report loudness aversion via a hyperacusis questionnaire. These findings suggest that CND may be a potential trigger for loudness hypersensitivity.

## 1. Introduction

Hyperacusis is a debilitating auditory condition wherein sounds of moderate intensity are described as unbearably loud, aversive, or painful (Tyler *et al.*, 2014). Sound intolerance complaints in hyperacusis are theorized to share a common root with maladaptive hyperexcitability (i.e., increased gain) in the central auditory system that can occur independent of cochlear hair cell damage (Auerbach *et al.*, 2014; Auerbach *et al.*, 2019; Herrmann and Butler, 2021). In fact, many patients with hyperacusis have clinically normal hearing thresholds, demonstrating that perceptual hypersensitivity may arise through peripheral damage that is undetected by standard threshold audiometry (Anari *et al.*, 1999; Brandy and Lynn, 1995; Sheldrake *et al.*, 2015; Urnau and Tochetto, 2011).

Likewise, rodents with primary cochlear neural degeneration (CND) exhibit paradoxically strong growth of neural responses along the central auditory neuroaxis at suprathreshold sound intensities (Asokan *et al.*, 2018; Chambers *et al.*, 2016; Hickox and Liberman, 2014; Qiu *et al.*, 2000; Resnik and Polley, 2017, 2021). This enhanced central gain is associated with the emergence of hyperacusis-like behavior in the animals, including elevated loudness perception (Auerbach *et al.*, 2019; Hickox and Liberman, 2014; Sun *et al.*, 2012) and sound aversion (Chen *et al.*, 2014; Manohar *et al.*, 2017). Although decades of research on acoustic injury in animal models links auditory deprivation and sensorineural hearing loss to heightened physiological and behavioral sound-evoked responses, a compelling link between peripheral processing deficits and central hypersensitivity in humans remains elusive.

Here, we examine whether variation in loudness perception and sound aversion in individuals with clinically normal audiograms is related to cochlear peripheral neural status. One challenge in addressing this question is that participants can vary in their subjective reporting of loudness due to implicit criteria for what qualifies as loud or soft. Individual biases in the mapping of sound intensity to perceptual labels could occur independent of peripheral neural status. Here, we controlled for the influence of individual biases by recruiting participants with asymmetric peripheral neural inputs between their two ears. In this way, the same participant's implicit bias in loudness reporting is maintained as a constant while sound is presented monaurally to ears selected for having pronounced asymmetries in peripheral neural status.

## 2. Methods

### 2.1 Participants

All procedures were approved by the institutional review board at Massachusetts Eye and Ear (MEE). Participants were recruited from a database of 165 healthy adults (aged 18–63 years) who had participated in prior research that aimed to identify peripheral markers of CND (Mepani *et al.*, 2020). All individuals in the database had normal audiometric thresholds from 0.25 to 8 kHz [ ≤25 dB hearing level (HL)], no interaural threshold asymmetry, and normal middle ear function.

**Fig. 1.**
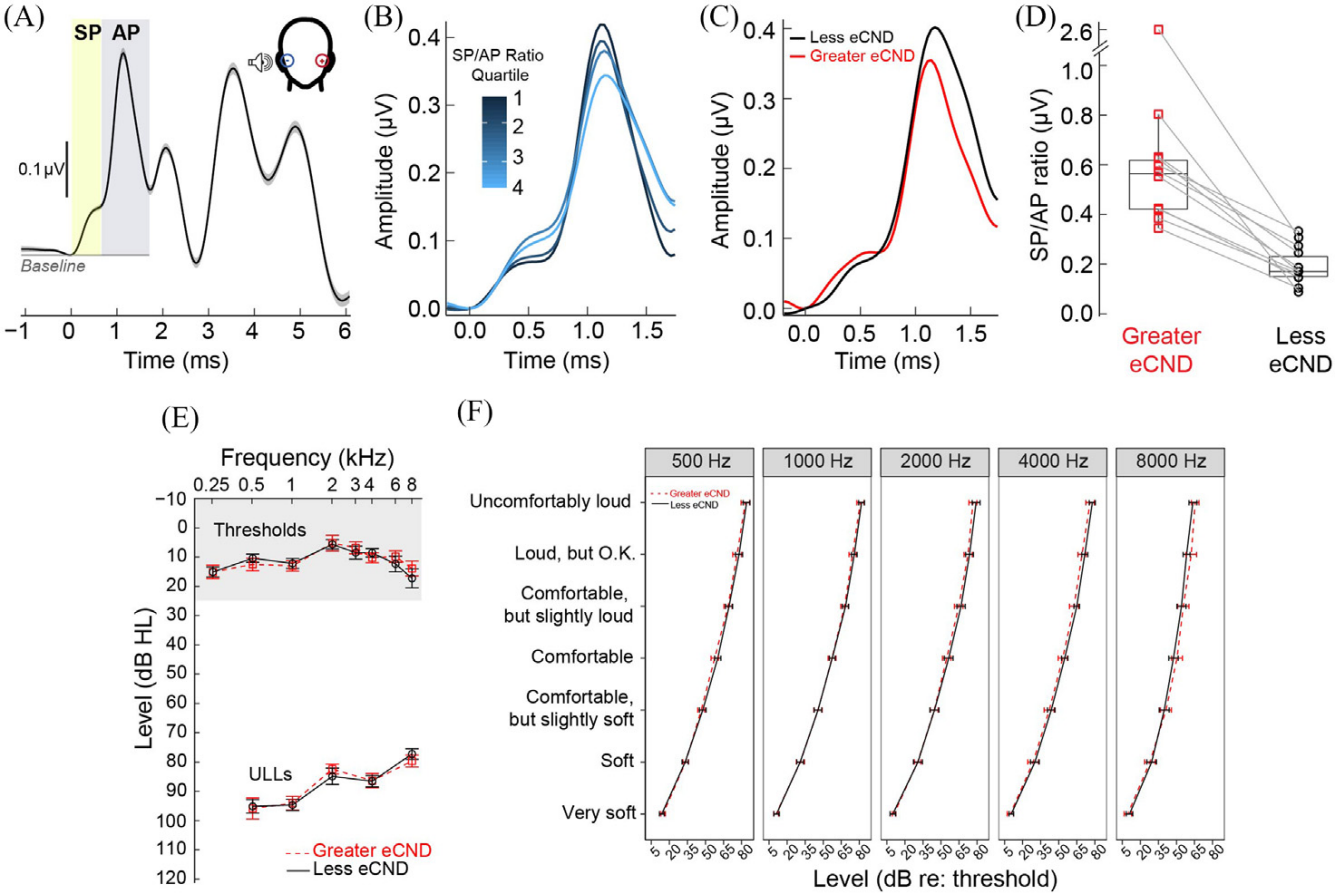
Participant selection and conventional clinical estimates of hearing sensitivity. (A) Mean [±1 standard error (SE)] electrocochleography (ECochG) waveforms from 245 ears with normal audiograms (Mepani *et al.*, 2020); (B) ECochG data were divided into four quartiles based on the magnitude of the ratio of the summating potential (SP) amplitude to the action potential (AP) amplitude. (C) Mean SP and AP waveforms from the 12 individuals who participated in the present study. (D) SP/AP ratios from each ear of the 12 participants. (E) Mean (±1 SE) audiometric thresholds and uncomfortable loudness levels (ULLs), with the region of normal hearing shaded in gray. (F) Mean (±1 SE) categorical loudness scaling data.

Figure 1 outlines the participant identification process for the current study. The database contained ECochG (Electrocochleography) recordings from 245 ears [Fig. 1(A)]. ECochG was collected using a horizontal electrode montage with the ground on the forehead at midline, one gold foil ER3–26A/B tiptrode as the inverting electrode, and another as the noninverting electrode in the opposite ear [Fig. 1(A), inset]. Stimuli were 100-μs clicks delivered at 125 peak sound pressure level (pSPL) in alternating polarity at a rate of 9.1 Hz. Electrical responses were amplified 1 × 10^5^ times, averaged across 2000 sweeps, and digitally filtered with a 10–3000 Hz passband. Additional details of the ECochG recordings are outlined in Mepani *et al.* (2020).

The ECochG data from all 245 ears were divided into four quartiles based on the magnitude of the SP to AP amplitude ratio [SP/AP ratio; Fig. 1(B)]. The AP was defined as the difference between baseline and the maximum waveform value between 1 and 2 ms post-stimulus onset. The SP peak was defined as the highest inflection point preceding the AP. Individuals with highly asymmetric SP/AP ratios between their two ears (i.e., an interaural SP/AP ratio asymmetry) were invited to participate in the present study [Figs. 1(C) and 1(D)]. An asymmetric SP/AP ratio was defined as having two ears with an SP/AP ratio in separate, non-adjacent quartiles of the distribution (e.g., quartiles 1 and 4, quartiles 2 and 4, or quartiles 1 and 3).

Of the 20 individuals who met these criteria, 12 provided written informed consent to participate and completed the full study (four female, mean age = 37.8 years, range = 21–60 years). Prior to testing, eligibility was confirmed via videoconference by screening for cognitive function [Montreal Cognitive Assessment (MOCA) > 25 for inclusion], tinnitus presence and severity [tinnitus reaction questionnaire (TRQ) < 72 for inclusion], use of assistive hearing technology (participants did not use cochlear implants, hearing aids, or FM assistive devices), and English proficiency (participants were conversationally fluent in English).

### 2.2 Remote behavioral testing

#### 2.2.1 Hardware, software, and logistics

Participants completed a battery of psychoacoustic tests and questionnaires remotely over a period of 2 weeks. We mailed a tablet computer (Surface Pro 2, Microsoft, Redmond, WA) and calibrated circumaural headphones (AE2, Bose, Framingham, MA) to the participants' home address. Tablets were programmed with custom software that was developed with the Unity game engine (United Technologies, Bellevue, WA) and scripted in C# (Microsoft), that has been previously used to assess a range of psychoacoustic abilities, including audiometric thresholds (Chen *et al.*, 2020; Whitton *et al.*, 2016), loudness discomfort (Chen *et al.*, 2020), and subjective hearing difficulties (Chen *et al.*, 2020; Parthasarathy *et al.*, 2020; Whitton *et al.*, 2016, 2017).

Prior to the experiment, participants received a virtual orientation where they were shown how to log into the tablet, open the testing software, and begin the tests. Instructions for completing each test were communicated through text and images in the software. Ambient noise levels were measured prior to each test using the tablet microphone. If ambient noise levels exceeded 60 dBA, the participant was locked out of the software, provided with a message about excessive noise levels in the environment, and instructed to find a quieter location to complete the testing. Participants were encouraged to take breaks as needed in between tests.

#### 2.2.2 Self-perceived sound aversion

To quantify each individual's self-perceived aversion to everyday sounds, participants completed a modified version of the hyperacusis questionnaire (mHQ) (Khalfa *et al.*, 2001; Khalfa *et al.*, 2002). The mHQ is recommended by the American Speech-Language-Hearing Association (ASHA) for the clinical identification, assessment, and management of hyperacusis (American Speech-Language-Hearing Association, 2018). Questions on the mHQ target elements of sound aversion such as avoidance (e.g., “Do you ever turn down an invitation or not go out because of the noise you would have to face?”), distress (e.g., “Do noise and certain sounds cause you stress and irritation?”), and fear (e.g., “Are you afraid of sounds that others are not?”).

#### 2.2.3 Audiometric testing

Participants completed a standard audiogram in both ears (Whitton *et al.*, 2016) to confirm that their hearing thresholds remained within normal limits and to set the levels for subsequent loudness perception testing. Pulsed pure tones (four 200-ms pulses, 5-ms rise-fall time) were presented at octave frequencies between 0.25 and 8 kHz and at 3 and 6 kHz. Participants indicated if they heard the tone by pressing a virtual response button after each stimulus presentation. Responses were considered hits if they occurred within a response window of 2.5 s. To track threshold, the level of the tone was decreased by 10 dB following hits and increased by 5 dB following misses. Threshold was defined as the lowest intensity level that elicited a hit response on two of three ascending runs, or three of six runs if concordance was not achieved after three runs.

We did not measure extended high-frequency (EHF) thresholds on the tablets. However, EHF thresholds (9–16 kHz) measured in these 12 participants during the Mepani *et al.* (2020) study were not statistically associated with interaural differences in the SP/AP ratio (paired *t*-test: *t*(11) = 0.59, *p *=* *0.59).

#### 2.2.4 Loudness perception testing

ULLs (Uncomfortable loudness levels), defined as the lowest intensity level that elicited a rating of “7—uncomfortably loud” on the Contour Test of Loudness Perception (Cox *et al.*, 1997), were assessed in both ears. A categorical loudness rating task was selected, as it is the type of metric that is used to assess ULLs in clinical settings. For each run, participants were presented with a pulsed pure tone (four 200-ms pulses, 5-ms rise-fall time) that began at 5 dB below the hearing threshold and ascended in 5-dB steps until the participant reported that the level was “uncomfortably loud.” Each run terminated as soon as the participant reported a judgment of uncomfortably loud. Participants completed four runs at each octave frequency between 0.5 and 8 kHz, and the ULL was computed as the median sound pressure level of uncomfortably loud responses across the four runs. The auditory dynamic range (DR) at each frequency was defined as the difference between the ULL and the audiometric threshold. The frequency-specific DR was used to customize the levels of subsequent loudness growth tests.

Loudness growth for pure tones (1, 2, and 4 kHz) was assessed using a cross-modality matching task (Stevens and Guirao, 1963), wherein the length of a virtual bar was adjusted to match the perceived loudness of a tone. Prior to each run, a reference tone was presented at an intensity level that was equivalent to the 50% point of the participant's DR at the corresponding frequency. While the participant listened to the reference tone, a virtual reference bar was displayed on the tablet at a length that was equivalent to 50% of the maximum possible bar length. The participant was informed that the length of the virtual reference bar matched the loudness of the reference tone. Following familiarization with the reference stimulus, tones were presented at various intensity levels in between the participant's threshold and ULL at the corresponding frequency, and participants were instructed to adjust the length of the virtual bar to match the loudness of the tones that they heard. The intensity of the tone varied randomly in 5-dB increments, with the minimum presentation level set to 5 dB above the participant's threshold and the maximum presentation level set to 5 dB below the participant's ULL at the corresponding frequency. This allowed us to assess loudness growth across everyone's unique, frequency-specific DR while ensuring that the tones were neither inaudible nor uncomfortable. Each intensity level was repeated four times, and the perceived loudness was quantified as the mean virtual bar length reported across the final three repetitions. The order of all behavioral testing was randomized across ears and frequencies.

#### 2.2.5 Statistical analyses

Data were analyzed in R version 4.1.0 (R Core Team, 2021) using the lme4 (Bates *et al.*, 2015), lmerTest (Kuznetsova *et al.*, 2017), MuMIn (Bartoń, 2020), and SIMR (Green and MacLeod, 2016) packages. Due to the nesting of ears within participants, linear mixed-effects models with random effects for participant were employed for all primary analyses. To minimize small sample estimation bias, all linear mixed-effects models were fit using restricted maximum likelihood (REML) parameter estimates (McNeish, 2017).

## 3. Results

We sought to quantify the relationships between auditory nerve status, self-perceived sound aversion, and the psychoacoustic perception of intensity in individuals with clinically normal audiograms and asymmetric peripheral function between their two ears. We predicted that variation in loudness perception and self-reported sound aversion would be partially explained by the degree of estimated cochlear neural degeneration (eCND). Specifically, because each participant had one ear with greater eCND than the other, we predicted that the ear with greater eCND (i.e., larger SP/AP ratio) would exhibit enhanced loudness perception when compared to the ear with less eCND (i.e., smaller SP/AP ratio).

We first assessed relationships between conventional clinical measures of loudness perception and eCND. Two separate linear mixed-effects models were constructed to evaluate the effect of eCND on ULLs and DR. By design, ears were classified as having either “greater eCND” (larger SP/AP ratio) or “less eCND” (smaller SP/AP ratio). We noted substantial variability in clinical ULLs [range 60.25–109.00 dB HL, mean (*M*)* *=* *87.69 dB HL, standard deviation (SD)* *=* *10.10 dB] and auditory DR (range 50–100 dB, *M *=* *77.47 dB, SD = 11.94 dB) across ears and frequencies, irrespective of eCND status. Both ULLs [*F*(1,98.64) = 58.36, *p *<* *0.001] and DR [*F*(1, 88.09) = 64.73, *p *<* *0.001] decreased significantly with increasing stimulus frequency between 0.5 and 8 kHz [Fig. 1(D)]. However, we did not observe significant associations between those single-point estimates of loudness perception and eCND [*p*s* *>* *0.05, Figs. 1(D) and 1(E)].

We next assessed the relationships between eCND, self-reported sound aversion, and loudness perception for intensities in between threshold and ULL. Loudness perception was quantified as the normalized virtual slider length that was reported for each stimulus intensity level during the cross-modality matching task. As the auditory DR varied on a participant-, ear-, and frequency-specific basis, we focused on the intensity levels (re: the audiometric threshold) that were common to all participants [range = 5–45 dB sensation level (SL)].

**Fig. 2.**
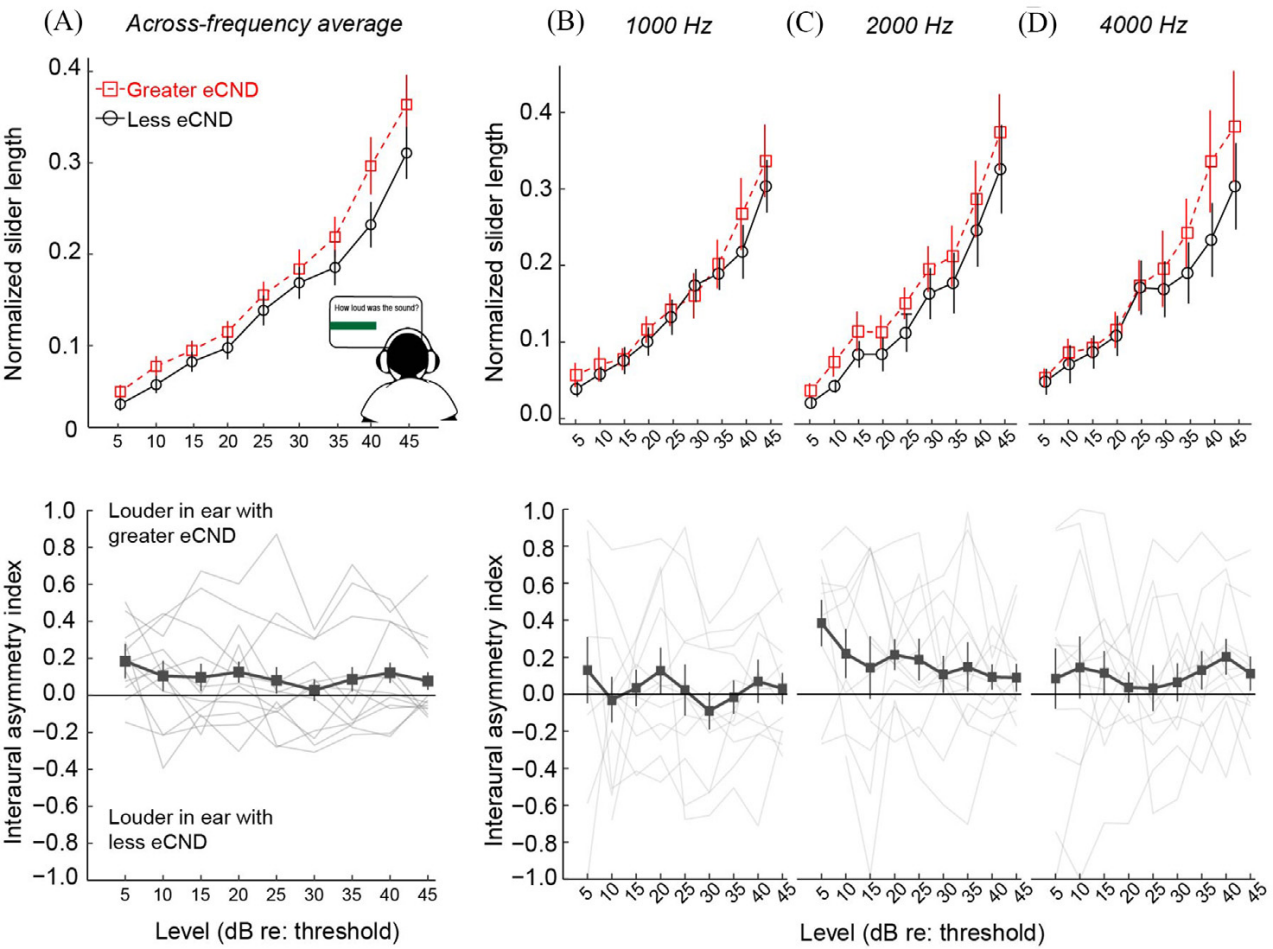
Loudness perception (estimated via cross-modality matching) for intensities in between threshold and the ULL as a function of eCND status. Top row: Loudness growth functions. Bottom row: Interaural asymmetry in loudness perception [interaural asymmetry index = (high SP/AP ratio – low SP/AP ratio)/(high SP/AP ratio + low SP/AP ratio)]; (A) data averaged across frequencies (Mean ±1 SE); (B) data for a 1000 Hz tone (Mean ±1 SE); (C) data for a 2000 Hz tone (Mean ±1 SE); (D) data for a 4000 Hz tone (Mean ±1 SE).

**Fig. 3.**
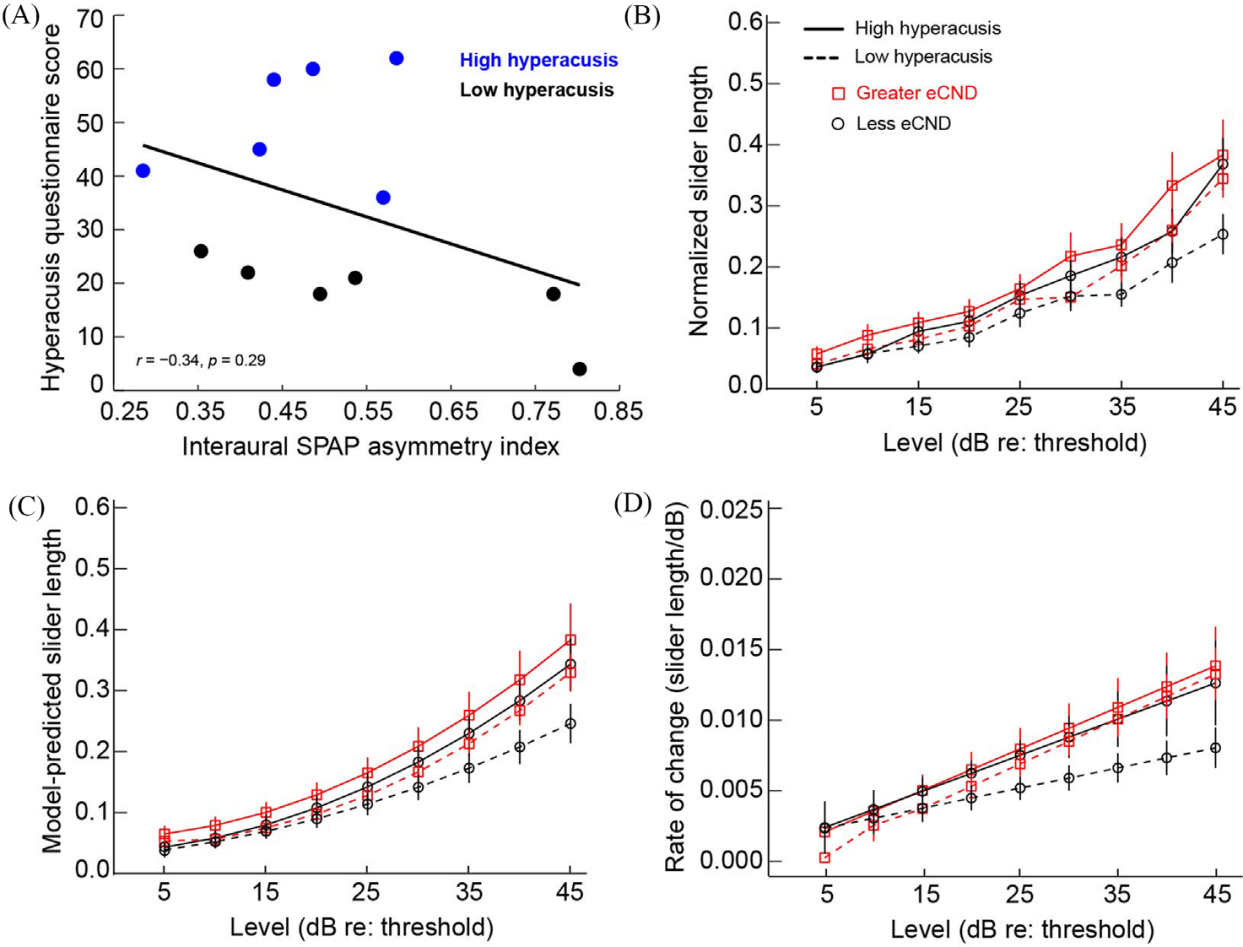
Relationships between self-reported sound tolerance, eCND status, and loudness perception (estimated via cross-modality matching). (A) Scores on the mHQ as a function of interaural asymmetry in SP/AP ratio. Blue points represent mHQ scores that fell above the group mean, and black points represent mHQ scores that fell below the group mean; (B) mean (±1 SE) loudness growth functions at 2 kHz as a function of mHQ score and eCND; (C) mean (±1 SE) model-predicted (polynomial fit) loudness growth functions at 2 kHz as a function of mHQ score and eCND; (D) mean (±1 SE) rate of change (slider length/dB) in loudness growth as a function of mHQ score and eCND. Rate of change was calculated as the derivative of the polynomial functions (C) at each sensation level.

**Fig. 4.**
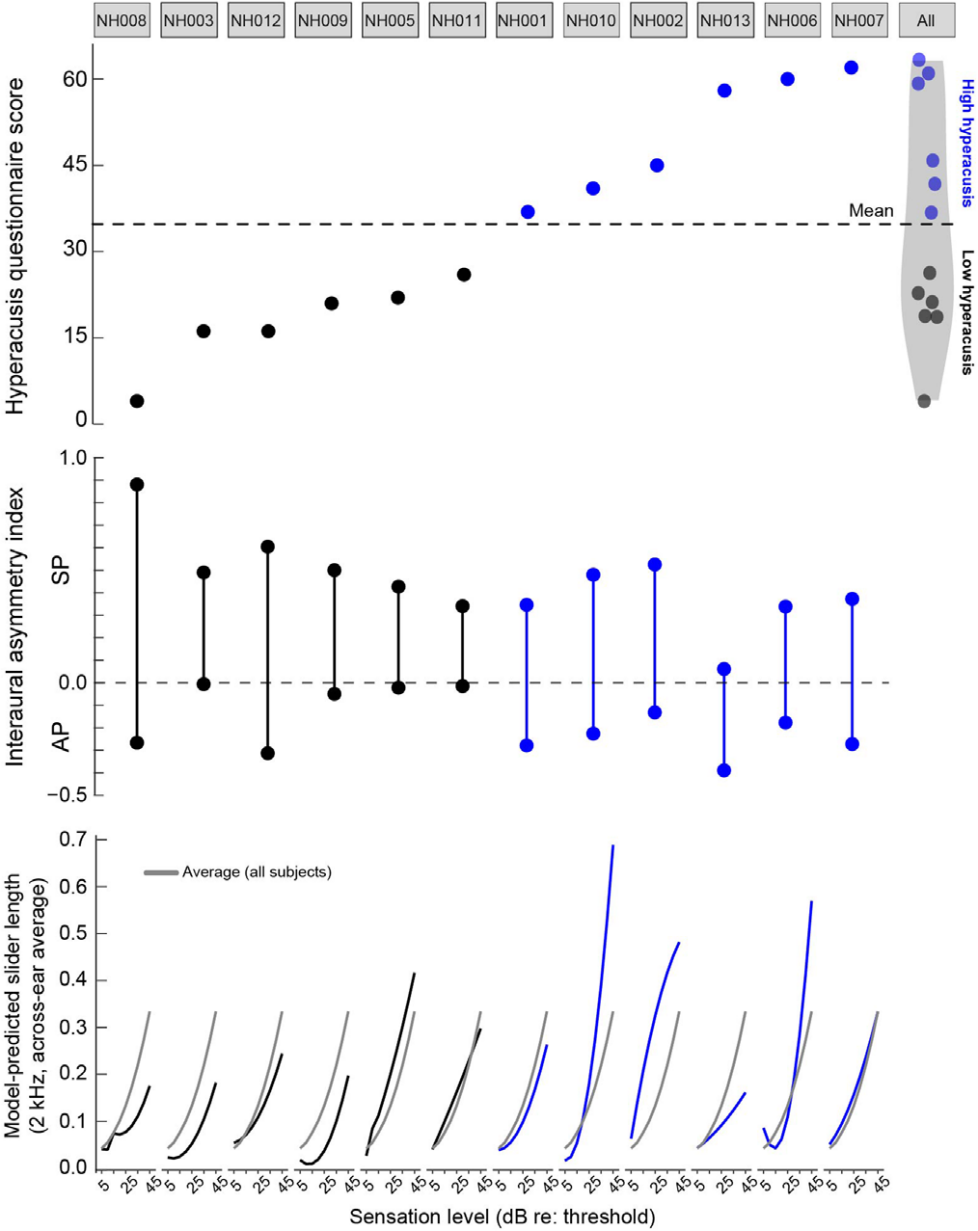
Individual data for each participant, ordered from best to worst self-reported sound tolerance (i.e., lowest to highest mHQ score). Top: mHQ score; middle: interaural asymmetry index for the SP and AP; bottom: across-ear average model-predicted loudness growth functions at 2 kHz.

Figure 2 shows loudness growth and the interaural asymmetry in loudness perception at each intensity level and frequency as a function of eCND status. Figure 3 shows the relationships between eCND status, self-reported sound tolerance, loudness perception, and the rate of loudness growth. The rate of loudness growth was quantified by fitting each loudness growth function with a second-order polynomial and calculating the derivative (i.e., rate of change in slider length/dB) at each point along the function. Figure 4 shows individual data for each measure.

Participants demonstrated substantial variability in eCND, self-perceived ability to tolerate sounds in daily life (mHQ scores), and loudness perception (Figs. 3 and 4). To determine the contribution of eCND to self-reported sound tolerance, we assessed the relationship between mHQ score and an eCND interaural asymmetry index [eCND interaural asymmetry = (high SP/AP ratio – low SP/AP ratio)/(high SP/AP ratio + SP/AP ratio)]. The relationship between eCND asymmetry and mHQ was weak and non-significant (Spearman *r*(10) = −0.34, *p *=* *0.29).

Since variation in eCND status was not significantly correlated with mHQ score, a linear mixed**-**effects analysis was performed to assess the combined effect of eCND and self-reported sound aversion on loudness perception for intensities in between threshold and ULL. To determine whether each predictor variable improved the model fit, an Akaike information criterion with a bias correction for small sample sizes (AICc) was used for parsimonious model selection (Hurvich and Tsai, 1989). First, we specified an empty model with participant as a random effect (AICc = −716.03). Next, we added fixed effects of frequency (levels: 1, 2, and 4 kHz) and intensity level (in dB re: threshold) (AICc = −1077.13). Finally, we consecutively added fixed effects terms for mHQ classification (low versus high hyperacusis scores) and eCND classification (low versus high SP/AP ratio). The model fit improved with the addition of each of the primary predictor variables. The model that resulted in the lowest AICc value (AICc = –1105.94) included fixed effects for frequency, intensity level, mHQ classification, eCND classification, and two interaction terms (mHQ × intensity level and mHQ × frequency).

Results from the linear mixed-effects analysis showed a significant main effect of eCND on normalized slider length [*F*(1, 624.01) = 12.63, *p *<* *0.001], wherein ears with greater eCND showed elevated loudness perception as compared to ears with less eCND (Fig. 2). Although the effect of eCND on loudness perception was small (*d *=* *0.20), the observed power to detect this effect was sufficient [78.80%; 95% confidence interval (CI): 76.13%–81.30%; Brysbaert and Stevens, 2018; Green and Macleod, 2016). The main effect of mHQ was not significant [*F*(1, 14.54) = 0.03, *p *=* *0.87]; however, significant interactions were observed for frequency and mHQ [*F*(2, 624.00) = 8.96, *p *<* *0.001] and intensity and mHQ [*F*(1, 624.03) = 7.90, *p *=* *0.005]. Specifically, loudness perception was enhanced binaurally at high sensation levels in participants with poor self-reported sound tolerance, and the effect was more pronounced for high-frequency stimuli (Figs. 3 and 4 show loudness growth data at 2 kHz).

## 4. Discussion

In the present study, we demonstrated that peripheral neural status and self-reported sound aversion each uniquely account for some variation in loudness perception amongst ears with clinically normal audiograms. These results suggest that changes in the psychoacoustic perception of intensity may be influenced by bottom-up contributions from the auditory periphery but that peripheral processing deficits may not fully account for the multifaceted nature of auditory hypersensitivity complaints.

Consistent with our ear-specific eCND findings, it is well-established in animal models that unilateral cochlear deafferentation triggers contralateral central overamplification and associated changes in loudness perception at suprathreshold sound intensities (Herrmann and Butler, 2021). At the same time, our subjective data are consistent with research showing that *monaural* attenuation of the acoustic environment leads to *binaural* changes in loudness perception that are nevertheless more pronounced in the deprived ear than in the control ear (Munro *et al.*, 2014). It is possible that peripheral auditory deprivation triggers tandem compensatory adaptions in the central nervous system that mediate affective responses to sound in addition to ear-specific psychoacoustic percepts (Chen *et al.*, 2014; Chen *et al.*, 2015; Manohar *et al.*, 2017).

The results of this study shed light on the role of peripheral mechanisms underlying complaints of elevated loudness perception and sound aversion that are commonly reported by patients with hyperacusis. From a clinical perspective, these findings highlight the importance of quantifying sound tolerance from multiple angles and across the entire auditory DR. Consistent with prior work (e.g., Sheldrake *et al.*, 2015), our data show that single-point clinical estimates of ULL are not sensitive to subtle differences in self-perceived sound tolerance. It is possible that more fine-grained assessments of loudness growth across the individual DR can better characterize systematic variation in behavioral loudness hypersensitivity (Brandy and Lynn, 1995).

We acknowledge the limitations imposed by the small sample size in the present study but note that focusing on a novel cohort of participants with asymmetric peripheral neural status helped to limit idiosyncratic factors in the psychoacoustic and electrophysiologic data. We also note that while we focused on a previously validated measure of eCND and its published participant cohort (Mepani *et al.*, 2020), it is possible that more recently developed estimates of eCND (Bramhall *et al.*, 2022; Mepani *et al.*, 2021) could better account for variation in perceptual auditory hypersensitivity. Future work will aim to objectively dissociate between complaints of loudness discomfort and affective sound processing at multiple stages of the central auditory pathway and in individuals with heterogeneous hypersensitivity phenotypes.
